# α-Mangostin Suppresses Melanoma Growth, Migration, and Invasion and Potentiates the Anti-tumor Effect of Chemotherapy

**DOI:** 10.7150/ijms.80940

**Published:** 2023-08-06

**Authors:** Yuxiu Xie, Chongwen Gong, Yun Xia, Yuhan Zhou, Ting Ye, Ting Mei, Hongxiang Chen, Jing Chen

**Affiliations:** 1 Cancer Center, Union Hospital, Tongji Medical College, Huazhong University of Science and Technology, Wuhan, China.; 2 Institute of Radiation Oncology, Union Hospital, Tongji Medical College, Huazhong University of Science and Technology, Wuhan, China.; 3 Department of Oncology, The Central Hospital of Wuhan, Tongji Medical College, Huazhong University of Science and Technology, Wuhan, China.; 4 Department of Plastic Surgery, Union Hospital, Tongji Medical College, Huazhong University of Science and Technology, Wuhan, China.; 5 School of Basic Medicine, Tongji Medical College, Huazhong University of Science and Technology, Wuhan, China.; 6 Department of Dermatology, Union Hospital, Tongji Medical College, Huazhong University of Science and Technology, Wuhan, China.; 7 Department of Dermatology, Huazhong University of Science and Technology Union Shenzhen Hospital, Shenzhen, China.

**Keywords:** melanoma, α-Mangostin, anti-tumor effect, chemotherapy, synergistic effect

## Abstract

**Purpose:** Melanoma is a highly malignant tumor, which metastasizes and has poor prognosis in late-stage cancer patients. α-Mangostin possesses pharmacological properties, including antioxidant, anti-infective, and anticarcinogenic activities. We investigated α-Mangostin effect on melanoma growth, migration, and invasion and its possible molecular mechanism.

**Methods:** Melanoma cells growth inhibition was determined by the colorimetric 4,5-dimethylthiazol-2-yl)-2,5-diphenyltetrazolium bromide reduction assay. Morphological changes of α-Mangostin-treated melanoma cells were evaluated by transmission electron microscopy and JC-1 staining. Cell apoptosis and cell cycle arrest were assessed by flow cytometry. The effect of α-Mangostin on tumor cells migration and invasion was observed by migration and invasion *in vitro* assay. Furthermore, the nude and C57BL/6 mouse subcutaneous melanoma models were used to evaluate the *in vivo* anti-tumor effect of α-Mangostin. Western blot and real time-PCR were performed to analyze the influence of α-Mangostin on some of the common signaling pathways in melanoma cell lines. Signaling pathways were further verified in dissected tumor tissues.

**Results:** α-Mangostin inhibited *in vitro* melanoma cells proliferation, migration, and invasion of melanoma cells, induced cell cycle arrest in G0/G1 phase, and caused mitochondrial swelling and membrane depolarization, whereas it effectively suppressed melanoma growth in xenografted mice. In addition, α-Mangostin potentiated the *in vitro* and *in vivo* anti-tumor effects of cisplatin both *in vitro* and *in vivo*. Mechanistically, α-Mangostin down-regulated expression of RAS protein and mRNA, as well as phosphorylation of PI3K in A375, B16F10, M14 and SK-MEL-2 cells. MITF protein and mRNA were inhibited only in M14 cells.

**Conclusion:** α-Mangostin suppresses melanoma cells growth, migration and invasion, and synergistically enhances the anti-tumor effect of chemotherapy, whose mechanism may be mediated through inhibiting Ras, PI3K and MITF.

## Introduction

Melanoma is a highly malignant tumor with multiple organ metastasis, highly increasing incidence, high mortality and poor prognosis.^1^ With the clinical success of immune checkpoint inhibitors, the progression free survival and overall survival of patients with advanced melanoma have been greatly extended. However, the objective clinical responses of immune checkpoint inhibitors lead by anti-PD-1 therapy is only 30% to 40%.^2^ *BRAF* and *MEK* inhibitors represent the standard first-line therapy *for BRAF*-mutated advanced melanoma*,* but BRAF gene mutation rate in Chinese population is less than 26%.^3^ Clinically, some patients have developed resistance to *BRAF* and *MEK* inhibitors, or have experienced serious drug-related adverse reactions.^4^, ^5^ Therefore, chemotherapy remains the main treatment for patients experiencing primary or acquired resistance to immune checkpoint inhibitors or BRAF-MEK inhibitor. However, overall response rate to traditional cytotoxic drugs in advanced melanoma is only 10% to 15%.^6^, ^7^ It is then necessary to evaluate new and more effective anti-melanoma drugs.

Mangosteen is a traditional medicine in Southeast Asia used to treat skin infections, wounds, fever and diarrhea.^8^ Its main bioactive components extracted from bark are α-Mangostin, β-Mangostin, γ-Mangostin, garcinone E and gartanin.^9^, ^10^ Studies have shown that drugs containing an oxanthone structure usually possesses antioxidant, anti-tumor, and anti-inflammatory properties.^11^, ^12^ It has been confirmed that α-Mangostin has anti-tumor effect on varies types of malignant tumors, such as colon, breast, prostate and pancreatic cancers.^13^, ^14^ It induces tumor cell cycle arrest, inhibits cell viability, promotes apoptosis and suppresses tumor invasion.^13^, ^15^, ^16^ We have previously completed the drug screening of microphthalmia-associated transcription factor (MITF) gene regulators in melanocytes in the Cancer Center and the Center for Dermatology of Massachusetts General Hospital of Harvard University. After screening more than 40 000 diverse natural extracts, α-Mangostin from Mangosteen bark extracts attracted our attention for its significant down-regulation of MITF expression.^17^ MITF is not only involved in the development, differentiation and functional regulation of pigment cells, but also constitutes an important cancer-related gene in melanoma cells. It also plays a key role in regulating the senescence, apoptosis, proliferation, and migration of melanoma cells.^18^-^20^

Taken together, the aim of the present study was to evaluate the effects of α-Mangostin on *in vivo* and *in vitro* melanoma cells growth, apoptosis, invasion, and migration and determine its synergistic effect with chemotherapeutic drugs and potential mechanism of anti-tumor effect. Results will provide a theoretical basis for new drug development and clinical application in melanoma.

## Material and methods

### Cell culture and treatment

The human melanoma cell lines A375 and M14 were cultured in DMEM culture medium (Gibco BRL, Gaithersburg, MD, USA), containing 10% fetal bovine serum (FBS), where the murine cell line B16F10 and the human melanoma cell line SK-Mel-2 were cultured in RMPI1640 culture medium (Gibco BRL), containing 10% FBS in a humidified incubator at 37 °C and 5% CO_2_. α-Mangostin (Sigma-Aldrich, St. Louis, MO, USA) was dissolved in DMSO in the concentration of 10 mmol/L and stored at 4°C, whereas Cisplatin (Sigma-Aldrich) and dacarbazine (Sigma-Aldrich) were dissolved in phosphate buffered saline (PBS) at 10 mmol/L and stored at -20°C until use. Paclitaxel (Selleck Chemicals, Houston, TX, USA) and vincristine (Selleck Chemicals, USA) were dissolved in DMSO at 10mmol/L and stored at -80°C.

### Proliferation assay

Cell proliferation was measured by the colorimetric 3- (4,5-dimethylthiazol-2-yl)-2,5-diphenyl tetrazolium bromide assay (MTT assay; Sigma-Aldrich). We incubated 5×10³ cells/well in 96-well plates and different drug concentrations for 24 h, 48 h and 72 h. To evaluate the synergistic inhibitory effect of α-Mangostin combined with chemotherapeutic drugs on cell proliferation, we used 10 nmol/L α-Mangostin plus different concentrations of chemotherapeutic drugs against melanoma cells. After treatments, MTT 4 h at 37°C, after which supernatant fluids were discarded, and formazan crystal were dissolved in DMSO (150 μL / well). Optical density (OD) values were then read in a microplate spectrophotometer (Tecan Group Ltd., Mannedorf, Switzerland) at 540 nm. The cell proliferation index was calculated according to the following formula: experimental OD value/ control OD value.

### Cell cycle flow cytometry analysis

Cells were incubated in 6-well plates and treated with 0, 5, 10 μmol/L α-Mangostin for 24 h, after which a single cell suspension was prepared using 0.25% trypsin. As a control group, 0 μmol/L α-Mangostin was composed of 0.05 % of DMSO and PBS. After washing in PBS three times, cells were fixed with 70% ethyl alcohol at -20 °C, overnight. Next, we washed the cells and incubated them with RNase for 30 min at 37 °C, after which 1mg/mL propidium iodide (PI, Beyotime Biotechnology, Jiangsu, China) was added to cells and incubated for 30 min in darkness. We then determined the percentage of each cell cycle phase by flow cytometry (BD Biosciences, Franklin Lakes, NJ, USA).

### Apoptosis flow cytometry analysis

For this assay, we incubated tumor cells in 6-well plates with 0 (0.2 % of DMSO in PBS), 5, 10 μmol/L concentrations of α-Mangostin for 24 h after which a single cell suspension was prepared using 0.25% trypsin. After washing in PBS three times, cells were suspended in 250 μL of binding buffer and stained with PI and Annexin V-FITC in darkness. Cell apoptosis percentages were determined by flow cytometry.

### Western blot

Cells were treated with 0 μmol/L (0.05 % of DMSO in PBS), 5 μmol/L α-Mangostin for 24 h, then cells were lysed with cell lysis buffer (RIPA buffer and 1% PMSF) and whole-cell lysates, and protein concentrations were quantified using the BCA reagent (Applygen Technologies Inc., Beijing, China). Proteins were separated in 10% sodium dodecyl sulfate-polyacrylamide gels and electro transferred to PVDF membranes. Membranes were then blocked with 5% bovine serum albumin (BSA) in PBS plus 0.1% Tween-20 for 1 h at room temperature (RT), probed with primary antibodies overnight at 4 °C, incubated with secondary antibodies for 1 h at RT, and developed with the ECL Advance Western blotting detection kit (GE Healthcare, Piscataway, NJ, USA), using the following antibodies: anti-β-actin (1:1000; Servicebio, Wuhan, China), HRP-conjugated affinipure goat anti-mouse IgG (1:5000; Servicebio) and anti-rabbit IgG (1:5000; Servicebio), anti-MITF (1:1000; Proteintech, Rosemont, IL, USA), anti-RAS (1:1000; Cell Signaling Technology Inc.), anti-RI3K (1:1000; Cell Signaling Technology Inc.), and anti-pRI3K (1:1000; Cell Signaling Technology).

### Real time-PCR

Total RNA from tumor cells was isolated using the Trizol reagent (Invitrogen, Carlsbad, CA, USA). cDNA was reverse transcripted by the RevertAid kit (Invitrogen) and amplified by the AceQ® Universal SYBR qPCR Master Mix (Vazyme, Nanjing, China) on a StepOnePlus Real-Time PCR System (Thermo Fisher Scientific, Walthman, MA, USA), using GAPDH gene expression as an internal control. The primer sequences we used are shown in **Supplementary Table 1**.

### Cell migration and invasion assays

A375, M14, SK-MEL-2 and B16F10 cells were treated with α-Mangostin for 24 h, after which cell density was adjusted to 2×10^5^ cells/mL with serum free culture medium, and 200 μL cell suspension were added into the upper chamber of Transwell culture system (Corning, NY, USA) with or without pre-coated Matrigel (Sigma Aldrich) for cell invasion or migration assay, respectively, whereas 500 μL of culture medium were added to the lower chamber. After incubation for 8 h at 37 ^o^C in an atmosphere of 5% CO_2_ in 95% air, Transwell chambers were fixed with 4% paraformaldehyde and stained with 0.1% crystal violet. Non-migrated cells on the upper side of the membrane were wiped off with a damp cotton swab and chambers were dried. Cells migrating to the lower surface were photographed under a microscope and counted in random three field. Experiments were repeated three times.

### Transmission electron microscopy

Cells treated with α-Mangostin were precipitated in 1.5 mL centrifuge tubes. After which 1 mL of 2.5% glutaraldehyde-0.1M phosphate buffer (pH 7.4) solution was carefully added along the tube wall. After fixing at 4 ^o^C for 24 h, samples were rinsed with 0.1 M phosphate buffer three times, and immersed in 1% osmic acid-0.1M phosphate buffer solution for 2 h at RT. Samples were then rinsed with 0.1 M phosphate buffer three times and dehydrated in 50%, 70%, 80%, 90%, 95% and 100% ethanol. After dehydration, samples were soaked in a mixture of acetone and embedding agent (1:1) overnight, followed by soaking in pure embedding agent overnight. Specimens were polymerized in an oven at 60 ^o^C for 48 h, and cut into 60 nm ultra-thin slices, after which they were immersed in a 2% uranium acetate saturated aqueous solution and lead citrate for 15 min, and dried at RT overnight. Cellular ultra-morphological structure was observed and photographed by electron microscopy (Field Electron and Ion Company, Hillsboro, OR, USA).

### Mitochondrial membrane potential analysis

Cells were incubated in 6-well plates and treated with 0 (0.1 % of DMSO in PBS), 5, and 10 μmol/L α-Mangostin for 24 h, after which a single cell suspension was prepared using 0.25% trypsin and suspended in 500μL of cell culture medium, stained with 500 μL of JC-1 working solution (freshlyprepared JC-1:dH20:dyeing buffer solution at a ratio of 1:1600:400 respectively, Beyotime Biotechnology, China) and incubated for 20 min at 37 °C. Cells were then washed with PBS twice and suspended with 1 mL PBS before evaluation by fluorospectro photometer (Eppendorf, Germany).

### In vivo experiment

The animal study was approved by the Animal Ethics Committee of Tongji Medical College, Huazhong University of Science and Technology. The A375 subcutaneous melanoma model was established using eight-week-old female nude mice. A375 cells suspension (1 × 10^6^ cells/100 μL in 1× PBS) was injected into the mouse right flank. In addition, B16F10 cells were trypsinized and washed with PBS twice before suspended in PBS at 5×10^6^/mL, after which 100 μL of this cell suspension were subcutaneously injected into the right flank of six-weeks-old female C57/BL mice. Seven days after implantation, α-Mangostin, cisplatin, and dacarbazine were intraperitoneally administered every two days. α-Mangostin was dissolved in a mixture of 20% anhydrous ethanol and 80% castor oil at a concentration of 2.4 mg/mL. Cisplatin and dacarbazine were dissolved in PBS at 0.9 mg/mL and 1.35 mg/mL, respectively. The following doses were applied per treatment group: 10 mg/kg α-Mangostin, 3 mg/kg cisplatin, 4.5 mg/kg dacarbazine. Mice in control group 1 received 0.1 mL PBS and 0.1 mL anhydrous ethanol-castor oil mix injections. Because DMSO may be toxic, mice in control group 2 (PBS + DMSO) were intraperitoneally administered with 0.1 mL of 2.5% DMSO. Mice weight and tumor volume were measured every two days. At the end of the treatment, mice were euthanized and entire tumors were removed and fixed with 10% paraformaldehyde for further experiments. Nude mice blood was obtained for biochemical analysis, and normal tissues such as heart, liver, spleen, lung and kidney were dissected for histopathological analysis. Animal experiments were accomplished under the protocols of the Hubei Provincial Animal Care and Use Committee, following the guidelines and ethical standard of the Animal Experimentation Ethics Committee of Tongji Medical College, Huazhong University of Science and Technology (Wuhan, China).

### Statistical analysis

Data represent mean ± SEM of triplicate determinations from three independent experiments. Statistical differences were determined by the student *t*-test and one-way ANOVA, using GraphPad Prism software (GrapPad Inc., San Diego, CA, USA).

## Results

### α-Mangostin inhibits melanoma cells growth in vitro

**Figure 1:**
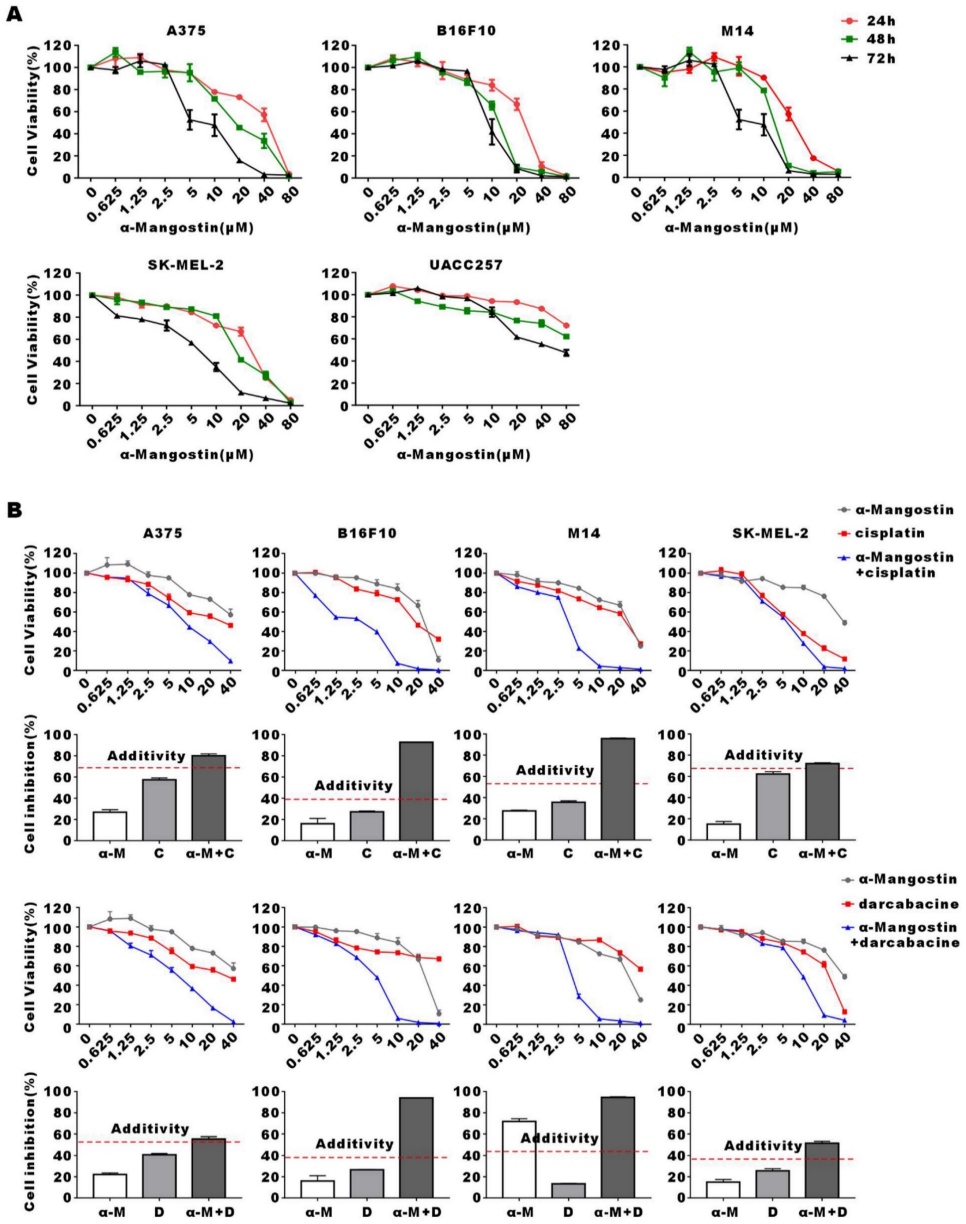
** Inhibitory effect of α-Mangostin and chemotherapeutic drugs on melanoma cells proliferation.** (A) Melanoma cells viability after treatment with different concentrations of α-Mangostin for 24 h, 48 h, and 72 h. (B) Melanoma cells viability after treatment with 10 μmol/L α-Mangostin and/or different concentrations of cisplatin/darcabacine for 24 h. Data represent the mean ± SEM (n = 5).

Human melanoma cells A375, M14, SK-MEL-2, UACC257 and mouse melanoma cell line B16F10 were treated with increasing concentrations of α-Mangostin for 24 h, 48 h and 72 h, after which cell viability was evaluated by the colorimetric MTT reduction assay (**Fig. 1A**). We showed that viability of α-Mangostin-treated A375, M14, SK-MEL-2, UACC257 and B16F10 cells was significantly (*p* < 0.05) reduced in a time- and concentration-dependent manner. The IC_50_ of cells treated with α-Mangostin in each group is shown in **Table 1**. To evaluate the synergistic antitumor effect of α-Mangostin and chemotherapeutic drugs in vitro, cells were incubated with α-Mangostin or/and commonly used clinical chemotherapeutic drugs (cisplatin, dacarbazine, paclitaxel and vincristine) for 24 h. Viability of cells incubated with α-Mangostin and chemotherapeutic drug alone or in combination was determined by colorimetric MTT reduction assay, after which the proliferation inhibition rate (IR), the synergistic index (CI_50_) and addition inhibition rate (E_bliss_) were calculated. CI_50_ < 1 and E_bliss_ < IR of combination indicates that the chemotherapeutic drug has a synergistic effect when combined with α-Mangostin. As shown in **Figure 1B, Supplementary Figure 1, and Table 2**, CI_50_ of α-Mangostin + cisplatin and α-Mangostin + dacarbazine were lower than 1, and the E_bliss_ of the combined treatment groups were lower than IR, which suggested that α-Mangostin potentiated the anti-tumor effect of cisplatin and dacarbazine. The CI_50_ of paclitaxel and vincristine combined with α-Mangostin was > 1 and IR was higher than that of E_bliss_. Thus, these results indicated that α-Mangostin had strong anti-tumor effect *in vitro* and had synergisticy effect with cisplatin and dacarbazine.

### α-Mangostin induces apoptosis and G0/G1 arrest of melanoma cells

**Figure 2:**
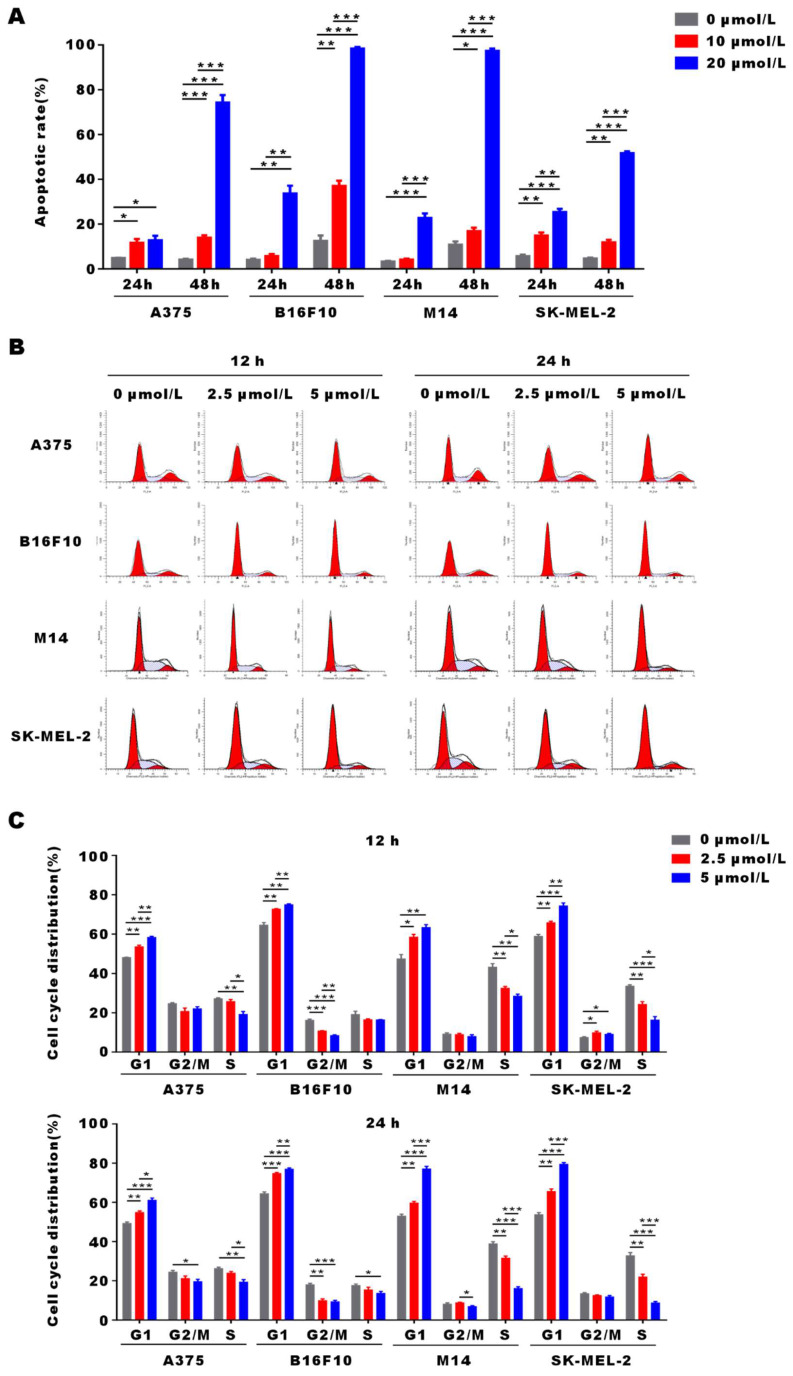
** α-Mangostin induces apoptosis and G0/G1 cell cycle arrest in melanoma cells.** (A) Histograms of cell apoptosis rates of melanoma cells treated with α-Mangostin. (B) Cell cycle distribution after treatment with α-Mangostin. (C) Statistical analysis data from (B). Data represent the mean ± SEM (n = 3).

To demonstrate α-Mangostin anti-tumor mechanism, we evaluated apoptosis and cell cycle distribution in α-Mangostin-treated melanoma cells by flow cytometry. As shown in **Figure 2A**, apoptosis of A375, B16F10, M14 and SK-MEL-2 cells was significantly induced after treatment with 20 μmol/L α-Mangostin for 48 h, and their apoptosis levels were increased by 70.1%, 85.92%, 86.49% and 47.1%, respectively (p < 0.001). In addition, 2.5 μmol/L α-Mangostin caused accumulation in G0/G1 phase in A375 (the percentage of G0/G1 was 48.12±0.04% vs 53.52±1.32%, p < 0.01), B16F10 (64.61 ±3.10% vs 72.71 ±0.13%, p < 0.01), M14 (47.45 ±9.31% vs 58.52 ±3.54% cognate p < 0.05), and SK-MEL-2 (58.94 ±1.51% vs 65.86 ±0.97%, p < 0.01) cells (**Figs. 2B and 2C**), whereas 5 μmol/L α-Mangostin induced percentages of G0/G1 of 58.35 ±0.50%, 75.00 ±0.28%, 63.46 ±3.61% and 74.43 ±4.06%, respectively. Thus, α-Mangostin induced the apoptosis and G0/G1 arrest to inhibit *in vitro* melanoma cells.

### α-Mangostin activates mitochondrial membrane depolarization in melanoma cells

**Figure 3:**
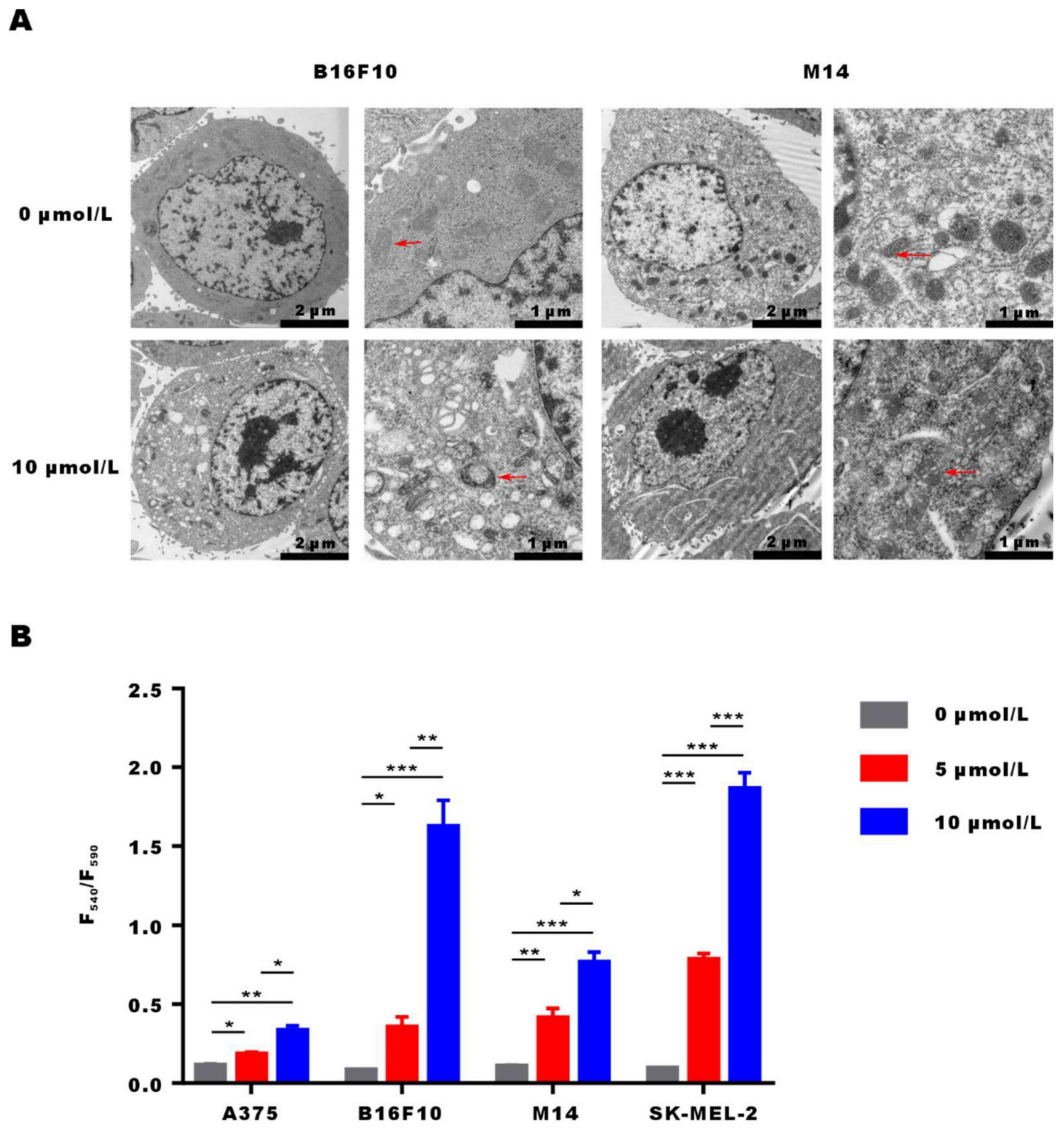
**α-Mangostin induces changes in the mitochondrial morphology and membrane potential.** (A) Representative images of ultrastructural changes in mitochondria. (Red arrows indicate mitochondria). (B) Mitochondrial membrane potential of melanoma cells treated with α-Mangostin.

After incubation of M14 and B16F10 cells with α-Mangostin for 3 h, they were fixed, embedded and stained, and cellular ultrastructural changes were observed by transmission electron microscopy** (Fig. 3A)**. Results showed that overall morphology and cell membrane of M14 and B16F10 cells were not affected by α-Mangostin. However, cell nuclei showed regional dense granular material and mitochondria evidenced obvious swelling, a vacuole-like structure, and disappearance or discontinuity of their crest structure in the α-Mangostin group, whereas mitochondria membrane was altered in the control group. We further examined the effect of α-Mangostin on mitochondrial membrane potential of melanoma cells. As shown in** Figure 3B**, the F540/F590 ratio of A375, B16F10, M14 and SK-MEL-2 cells treated with 10 μmol/L α-Mangostin significantly (p < 0.01, 0.001, 0.001, 0.001) increased (0.33 ± 0.05, 1.62 ± 0.29, 0.76 ± 0.11 and 1.86 ± 0.18, respectively), which showed that α-Mangostin significantly decreased the cellular mitochondrial membrane potential. Thus, these results suggest that α-Mangostin induced mitochondrial swelling and membrane depolarization of melanoma cells.

### α-Mangostin suppresses melanoma cells migration and invasion

**Figure 4:**
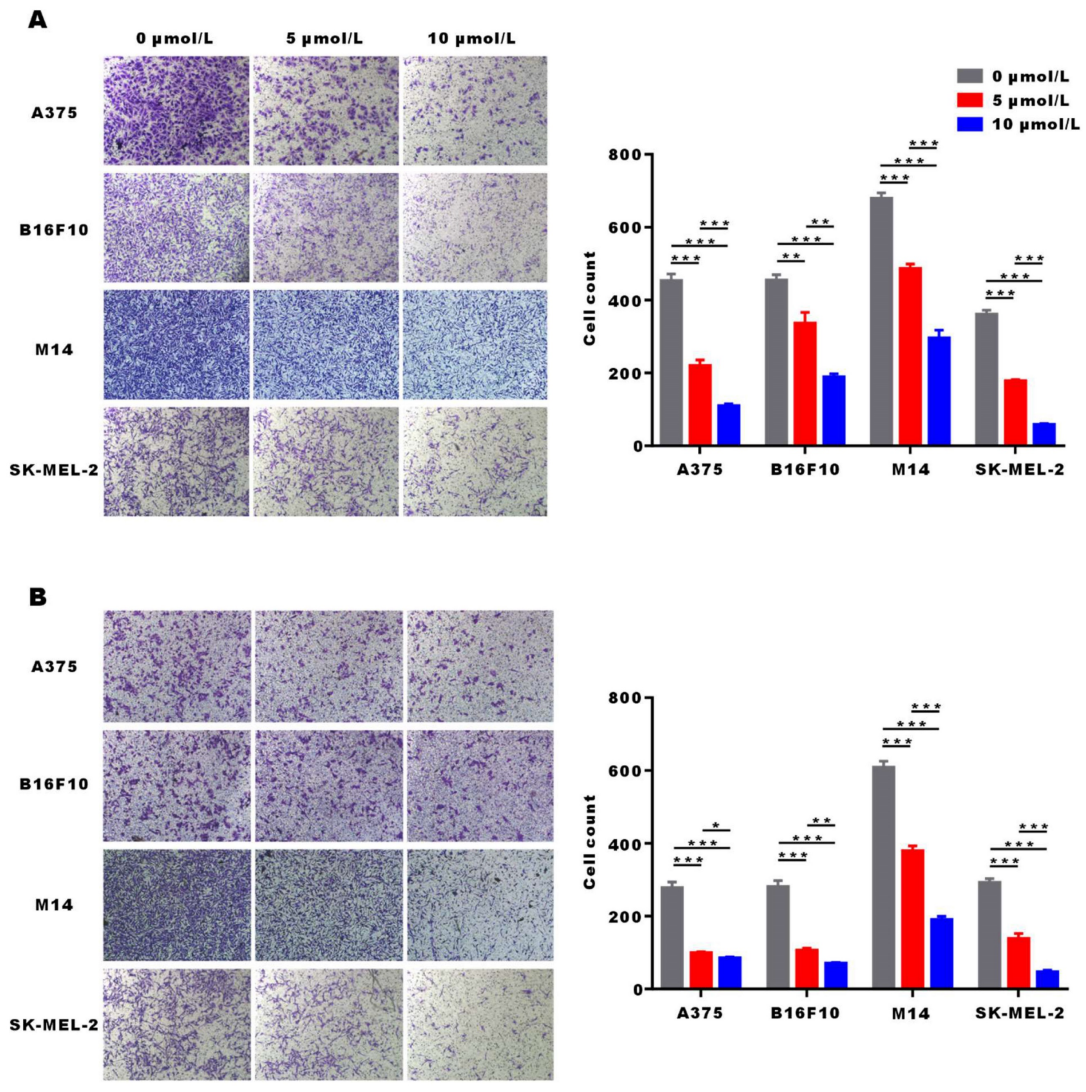
** α-Mangostin suppresses the migration and invasion potential of melanoma cells.** (A) Representative images and statistical analysis graph showing α-Mangostin inhibition of melanoma cells migration. (B) Representative images and statistical analysis graph showing α-Mangostin inhibition of melanoma cells invasion. Data represent the mean ± SEM (n = 3).

The migration and invasion potential of tumor cells is the key factor for distant metastasis and local invasion of melanoma. Transwell migration and invasion assays showed that the number of A375, M14, SK-MEL-2 and B16F10 cells entering the lower chamber significantly (p < 0.001) decreased after α-Mangostin treatment. This may indicate that α-Mangostin suppressed the invasion and migration of melanoma cells (**Figs. 4A and 4B**).

### α-Mangostin inhibits tumor cells growth and potentiate the anti-tumor effect of cisplatin in vivo

**Figure 5:**
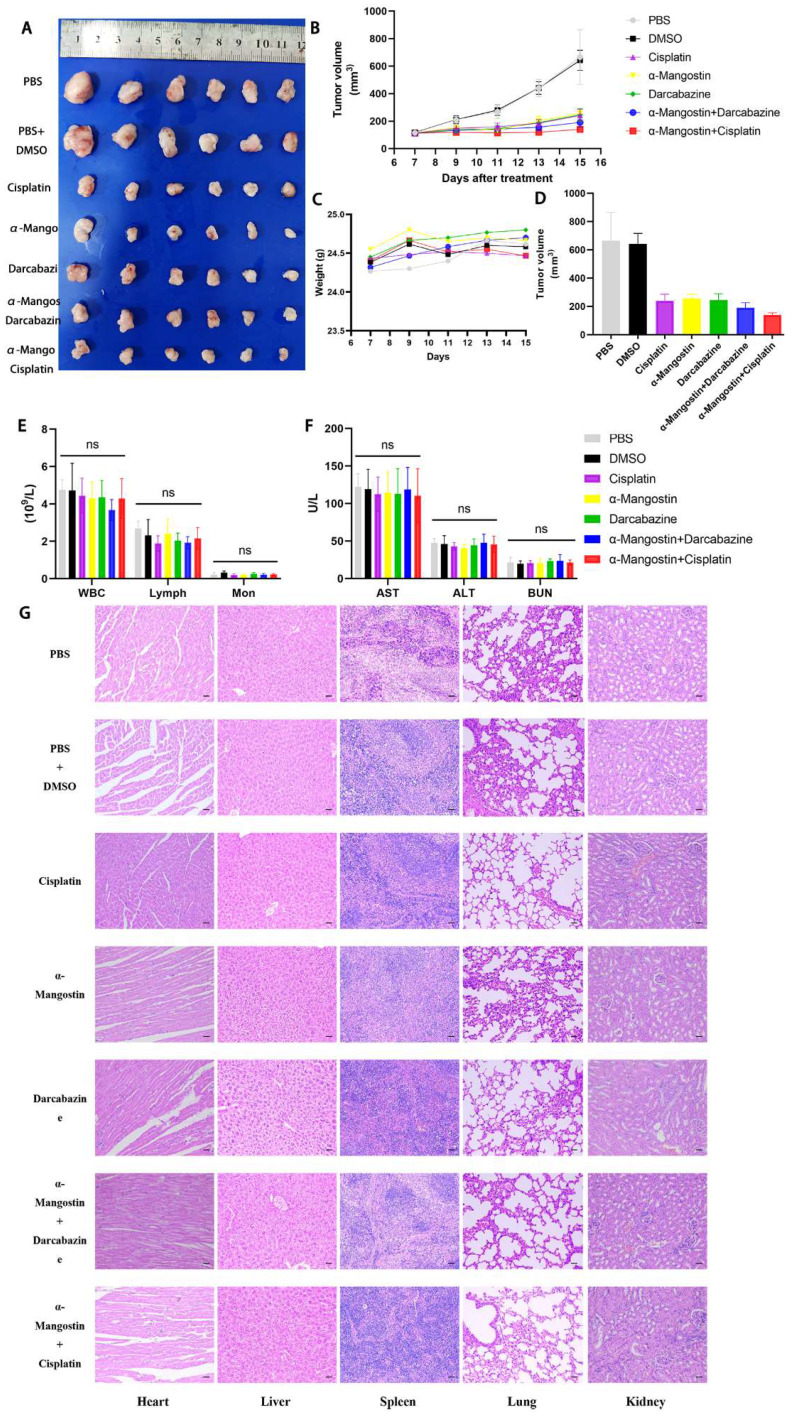
** α-Mangostin in combination with cisplatin/darcabacine inhibit A375 melanoma cells growth *in vivo*.** (A) Photograph of tumor dissected from nude mouse. (B) Tumor growth curves. (C) Weight of mice during treatment. (D) Tumor volume at day 8 after corresponding treatments, data represent the mean ± SEM. (n = 6). (E) (F) Hemanalysis and biochemical analyses on day 8 after treatment. Data represent the means ± SEM (n = 3). (G) Micrographs of H&E staining of major organs; the scale bar indicates 50 μm.

To determine the effect of α-Mangostin on melanoma cells growth and its potential synergistic effect in combination with chemotherapeutic drugs *in vivo*, a xenotransplantation nude mouse model of subcutaneous melanoma was established and treated with α-Mangostin, cisplatin, dacarbazine, cisplatin + α-Mangostin or dacarbazine + α-Mangostin, respectively. Results showed that α-Mangostin significantly (p < 0.05) reduced the tumor volume, as compared with the control group (**Figs. 5A, 5B, and 5D**). In addition, α-Mangostin in combination with chemotherapy (dacarbazine or cisplatin) significantly (*p* < 0.05) reduced tumor size, as compared with the control group. We did not observe differences in contraction between the combined treatment group (α-Mangostin + dacarbazine; α-Mangostin + cisplatin) and the monotherapy group (α-Mangostin; dacarbazine; cisplatin). However, α-Mangostin plus cisplatin treatment the lowest average tumor volume.

**Figure 6:**
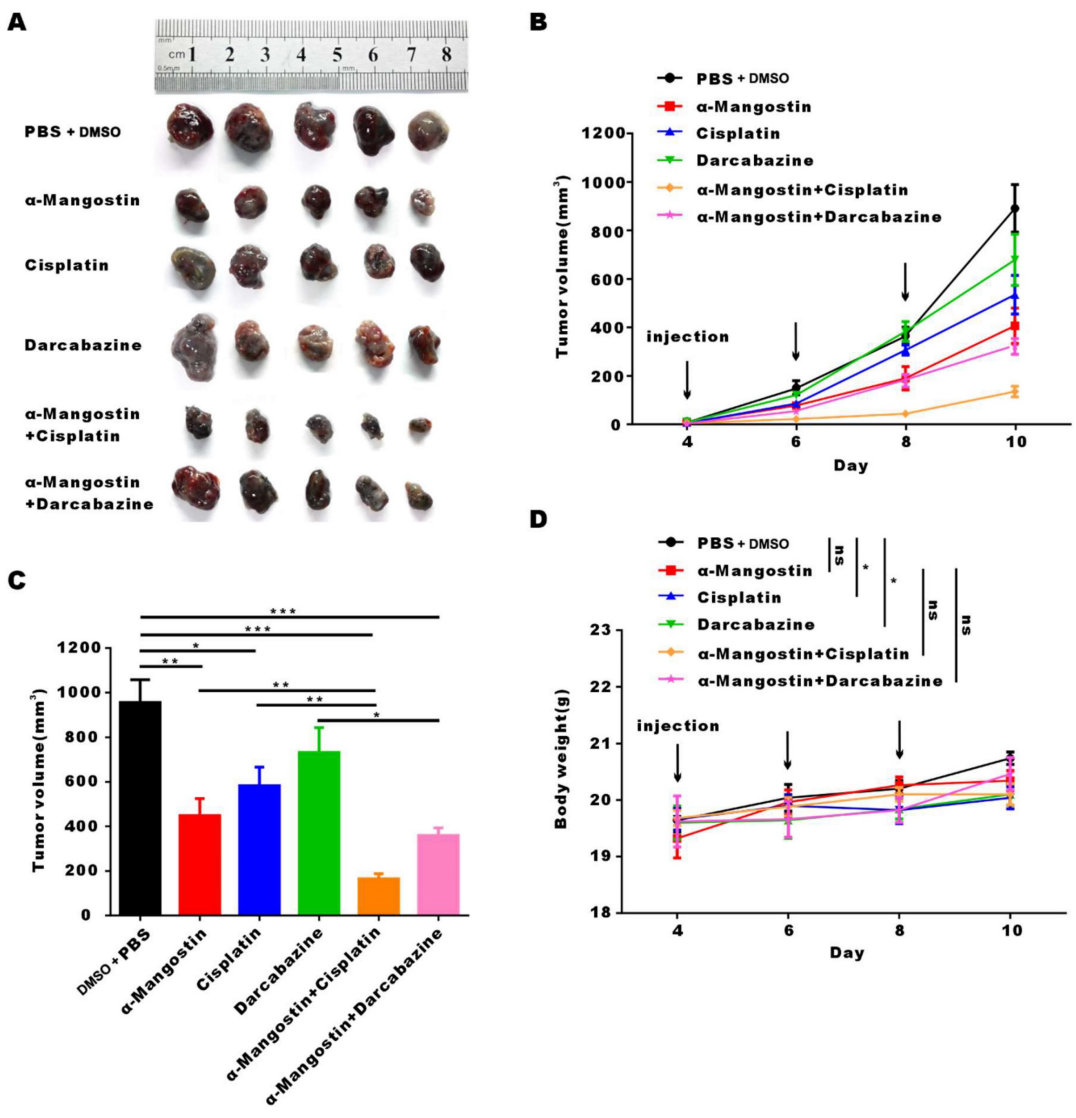
** α-Mangostin in combination with cisplatin/darcabacine inhibit B16F10 melanoma cells growth *in vivo*.** (A) Photograph of tumor dissected from six groups. (B) Tumor growth curves of six groups. (C) Statistical analysis data from (B). (D) Weight of mice during treatment. Data represent the mean ± SEM (n = 5).

To investigate the anti-tumor effect of α-Mangostin alone or in combination with chemotherapy in C57/BL mice with complete immune system, we established the B16F10 melanoma mouse model. Results showed that α-Mangostin significantly (*p* < 0.01) inhibited tumor volume (**Figs. 6A, 6B, and 6C**). Furthermore, tumor volume in the α-Mangostin + cisplatin group was significantly (*p* < 0.01) lower than that of the α-Mangostin and cisplatin groups. Tumor volume of the α-Mangostin + dacarbazine group was also lower than that of the dacarbazine group but we did not observe significant differences as compared with the α-Mangostin group. These results indicated that α-Mangostin exerted synergistic anti-tumor effect in combination with cisplatin *in vivo*.

We recorded changes of body weight during treatments to evaluate their safety. Results showed no statistical differences between the α-Mangostin group vs the control group, the α-Mangostin + dacarbazine group vs the dacarbazine group, and the α-Mangostin + cisplatin group vs the cisplatin group (**Figs. 5C and 6D**), indicating that α-Mangostin injection did not alter mice body weight. Furthermore, on the 8th day after drug treatment, all drug-treated groups, and the PBS or the DMSO groups showed similar numbers of white blood cells, lymphocytes, and monocytes (**Fig. 5E**). Biochemical analysis results did not show significant differences in blood glutamic pyruvic transaminase (GPT), aspartate aminotransferase (AST), and urea nitrogen (BUN) levels between the treatment group and the PBS group or the DMSO group (**Fig. 5F**).

HYPE staining sections of heart, liver, spleen, lung, and kidney did not show organic pathological damages in each group (**Fig. 5G**). Taken together these results demonstrated that α-Mangostin alone or in combination with chemotherapy is biocompatible and safe.

### α-Mangostin suppresses RAS and the phosphorylation of PI3K

**Figure 7:**
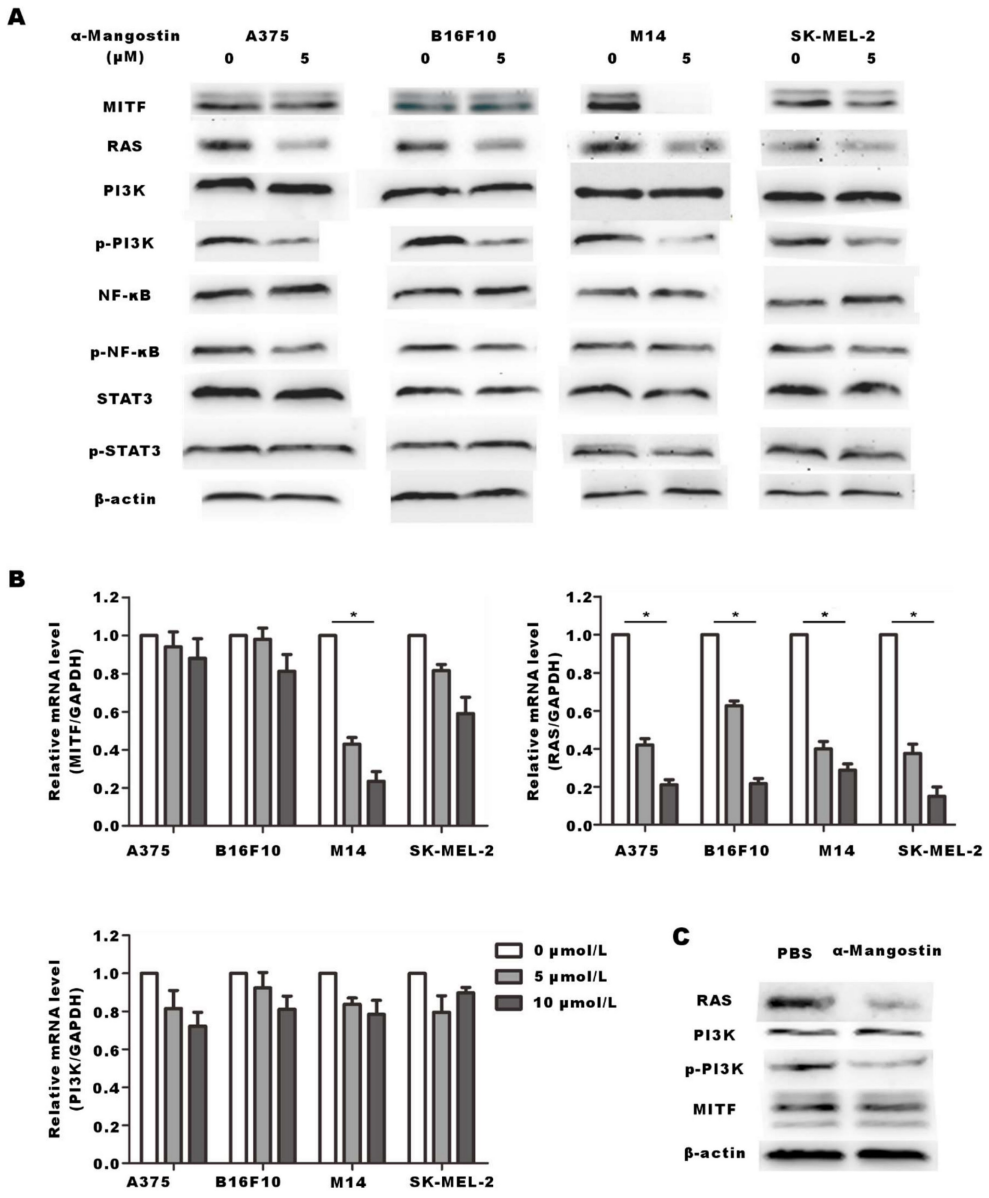
** α-Mangostin suppresses RAS and the phosphorylation of PI3K.** (A) Western blot analysis of MITF, RAS, PI3K, p-PI3K, NF-κB, p-NF-κB, STAT3, and p-STAT3 of melanoma cells after treatment with α-Mangostin. (B) mRNA expression of MITF, RAS, and PI3K in melanoma cells, after treatment with α-Mangostin. (C) Western blot analysis of MITF, RAS, PI3K and p-PI3K in tumor tissue after α-Mangostin injection.

We performed Western blotting and real time-PCR to explore the signal pathway related to the anti-proliferation effect induced by α-Mangostin. MITF is the key gene that controls the production of the tyrosinase family, which induces melanin synthesis. Studies have shown that RAS/PI3K/Akt/mTOR signal transduction axis plays a core role in promoting tumor cell growth, proliferation and survival by inhibiting apoptosis. RAS/PI3K/Akt/mTOR signal pathway is one of the important cellular signaling pathways that control the expression and nuclear export of MITF. 21 Results showed that RAS and p-PI3K expression in α-Mangostin-treated A375, B16F10, M14 and SK-MEL-2 cells were significantly down-regulated (**Figs. 7A and 7B**). Real time-PCR assay also showed that mRNA expression of RAS was down-regulated, whereas mRNA expression of PI3K was not altered. These results indicated that α-Mangostin suppressed the expression of RAS and the phosphorylation of PI3K. We have previously reported that α-Mangostin significantly down-regulated MITF expression in melanocytes. It well known that MITF may regulate apoptosis, proliferation, and migration of melanoma cells. We detected the expression of MITF in A375, B16F10, M14, and SK-MEL-2 cells. However, our results evidenced that α-Mangostin only down-regulated the expression of MITF protein and mRNA in the human melanoma cells M14. We then extracted total protein from tumor tissues, as mentioned in the *in vivo* experiment, and detected the expression of RAS, MITF, and phosphorylation of PI3K. RAS expression and PI3K phosphorylation in the α-Mangostin group were significantly lower than those in the control group, whereas MITF and PI3K expression was not significantly alter (**Fig. 7C**). It was further demonstrated that α-Mangostin may down-regulate the expression of RAS and PI3K phosphorylation* in vivo*.

## Discussion

Immunotherapy and *BRAF* and *MEK* inhibitors have improved the survival rate of melanoma patients., However, a marginal number of patients have completely recovered but many patients have primary or acquired drug resistance.^6^ Chemotherapy is still an important treatment strategy for advanced melanoma patients who fail immunotherapy or targeted therapy. Serious adverse reactions such as hepatotoxicity, nephrotoxicity and myelosuppression also limit the use of chemotherapeutic drugs. Developing new drugs with low toxicity, high efficiency and synergistic effect with other drugs is a challenging endeavor in the treatment of melanoma. In the present study, we demonstrated the anti-melanoma effect of α-Mangostin *in vitro* and *in vivo*, and its potentiating effect in combination with cisplatin.

Uncontrolled cell proliferation and blocked apoptosis are the basic biological characteristics of malignant melanoma cells.^22^, ^23^ In the present study, we showed that α-Mangostin inhibited cell proliferation and induced melanoma cells apoptosis. Abnormal cell cycle progression is the key factor leading to malignant cell proliferation.^24^ We further found that α-Mangostin induced G0/G1 phase arrest to inhibit the melanoma cells proliferation. Regarding cellular ultrastructure, α-Mangostin caused mitochondria swelling, disappearance or discontinuity of crest structure, and vacuole-like structure in melanoma cells. Furthermore, mitochondrial membrane potential showed that the F540/F590 ratio significantly increased, indicating that such a potential decreased, which may cause mitochondrial edema and destruction. Cell migration and invasion potential makes melanoma prone to systemic metastasis and local invasion, which is the main cause of poor prognosis and death. Using the Transwell chamber, we found that the proportion of melanoma cells migrating to the lower chamber significantly decreased after α-Mangostin treatment, thus indicating that α-Mangostin inhibits melanoma cells migration and invasion. *In vivo* studies also confirmed that α-Mangostin inhibited the growth and tumor volume of subcutaneously transplanted melanoma in nude and C57BL/6 mice.

We further studied the synergistic effect of α-Mangostin in combination with the chemotherapeutic drugs, dacarbazine and cisplatin. Dacarbazine is the earliest chemotherapeutic drug approved by FDA for the treatment of advanced melanoma, however, a number of clinical trials have shown that its overall effective rate is 13.4%, and the complete remission rate is very low.^25^-^27^ We observed that the synergistic index CI_50_ of α-Mangostin and dacarbazine in A375, B16F10, M14, and SK-MEL- 2 cells was < 1, whereas the addition inhibition rate E_bliss_ was lower than the IR of the combination, which confirmed the synergistic anti-tumor effect of α-Mangostin and dacarbazine *in vitro*. However, we did not find a statistical difference in tumor volume between the α-Mangostin + dacarbazine group and the α-Mangostin group, which might be related to the lower dose gradient of dacarbazine injection shown in previous studies, the therapeutic effect of α-Mangostin in combination with dacarbazine *in vivo* needs further investigation.

The effective rate of single cisplatin injection in melanoma is only 10% to 20%. In addition, it may cause severe side effects, including nephrotoxicity, ototoxicity, neurotoxicity, vomiting and myelosuppression.^28^ In the present study, we demonstrated that α-Mangostin potentiated the anti-tumor effect of cisplatin on melanoma *in vitro*. In the immune-deficient athymic nude mouse model of subcutaneous tumor transplantation, cisplatin in combination with α-Mangostin significantly inhibited melanoma growth, as compared with the control group, but there was no statistically significant difference between the combination treatment and cisplatin alone or α-Mangostin treatment. In the immunocompetent mouse C57 model, cisplatin in combination with α-Mangostin significantly inhibited melanoma growth, as compared with the monotherapy group. Studies reported that cisplatin recruited anti-tumor CD8+ cytotoxic T cells causing direct tumor cells totoxicity or growth inhibition.^29^ The anti-tumor effect of cisplatin depends in part on the integrity of the immune system, especially T cells. This also explained why our results showed that cisplatin combined in combination with α-Mangostin had a synergistic anticancer effect in immunocompetent mice but not in immunodeficient mice. Perez-Rojas*et al*. found that α-Mangostin has a protective effect on cisplatin-induced nephrotoxicity.^30^, ^31^ Taken together, we believed that the combination of α-Mangostin and cisplatin is of high clinical value to treat melanoma, which may not only enhance the inhibitory effect of cisplatin on tumor growth, but also reduce the occurrence of cisplatin-related side effects.

Furthermore, we investigated the possible anti-tumor molecular mechanism of α-Mangostin against melanoma. A previous drug screening showed that α-Mangostin significantly down-regulate MITF expression.^17^ MITF participates in the senescence, apoptosis, proliferation, cell cycle, migration and invasion of melanoma cells by regulating important signal molecules such as TBX2, CDK2, CDKN1A/B, CDKN2A, Bcl-2, C-Met and HIF-1 α.^20^, ^32^-^34^ In the present study, we evaluated whether α-Mangostin regulated the expression of MITF and biological events in melanoma. Results demonstrated that α-Mangostin inhibited A375, B16F10, M14 and SK-MEL-2 cells growth. However, down-regulation of MITF protein and mRNA expression was only observed in the human melanoma cell line M14 but not in other cell lines. Melanoma cells are divided into the MITF-dependent and MITF-independent two types.^35^ The basic expression of MITF was high in MITF-dependent melanoma cells such as M14 cells, and a significant decrease of MITF expression was observed after MITF inhibitors treatment, whereas it was difficult to observe obvious down-regulation of MITF in MITF-independent melanoma cells because of their low basic expression of MITF. Therefore, we hypothesized that the reason for α-Mangostin to down-regulate only the expression of MITF in M14 cells might be related to the different basic expression of MITF among these four types of melanoma cells, which requires further investigation.

In addition to MITF, we screened several potential molecules that mainly affect certain melanoma characteristics, including RAS, MITF, PI3K, NF-κB, and STAT3. RT-PCR and Western blot analysis indicated that α-Mangostin down-regulated protein and mRNA expression of RAS and PI3K phosphorylation in melanoma cells. It has been previously reported that RAS/PI3K/AKT/mTOR pathway plays an important role in tumorigenesis, regulating cell cycle distribution and angiogenesis, which is a potential target in melanoma therapy.^36^ Therefore, we further explored the regulatory effect in mice melanoma tissue and verified that *in vivo* α-Mangostin down-regulated the expression of RAS and the phosphorylation of PI3K. However, the specific molecular mechanisms of the upstream and downstream still need more assays. We will study the mechanism underlying the synergistic effect of α-Mangostin with chemotherapies, and clarify whether α-Mangostin mediates down-regulation of MITF for anti-tumor effect.

As a fat-soluble and small molecular weight drug, α-Mangostin is easy to obtain and develop as an agent with low toxicity. However, α-Mangostin has been observed to toxicity in high doses, and is poor water solubility, resulting in low bioavailability and hindering the use of α-Mangostin *in vivo*. In order to overcome its poor solubility, α-Mangostin may be stably loaded into nanomaterials or liposomes to achieve a more significant bioavailability. In addition, the results of this study found that α-Mangositin combined with chemotherapy drugs has a synergistic anticancer effect. Constructing a dual delivery system loaded with α-Mangostin and chemotherapy drugs would have better anti-cancer effects while reducing drug dosage and toxicity.

## Conclusion

We comprehensively investigated the anti-tumor effects of α-Mangostin on melanoma cells. It suppressed melanoma cell proliferation, migration, and invasion. and possessed synergistic *in vitro* and* in vivo* effect in combination with cisplatin. Regarding α-Mangostin anti-tumor mechanism, we found that this xanthone down-regulated RAS, p-PI3K and MITF expression. As a natural extract, α-Mangostin is easy to obtain and develop as an oral agent with low toxicity. Therefore, α-Mangostin is a potential anti-melanoma drug and chemotherapeutic sensitizer, which provides a theoretical basis for the development and clinical application of new drugs on melanoma patients.

## Supplementary Material

Supplementary figure and table.

## Figures and Tables

**Table 1:** IC_50_ of α-Mangostin on melanoma cells proliferation

IC_50_ (μmol/L)	24h	48h	72h
A375	34.8±7.4	19. 7±3.8	7.9±1.8
B16	23.1±3.2	11.6±1.1	9.4±0.8
M14	45.0±5.1	26.1±3.3	14.9±3.2
SK-MEL-2	22.1±3.7	18.6±2.4	5.2±0.8
UACC257	177.3±76.3	210.3±111.5	54.4±12.9

**Table 2:** Synergistic effect of α-Mangostin and chemotherapeutic drugs

Variable		A375		B16F10		M14		SK-MEL-2	
		IC_50_	IR*	IC_50_	IR*	IC_50_	IR*	IC_50_	IR*
Cisplatin	C	26.2	57.5	19.5	27.3	19.3	35.5	7.0	62.2
	C+α	8.6	80.2	2.1	92.8	3.3	95.6	5.2	72.1
	CI**_50_**	0.5		0.2		0.3		0.8	
	E_bliss_	68.9%		38.9%		53.3%		67.9%	
Dacarbazine	D	26.2	40.7	214.8	26.5	214.8	13.3	19.5	25.6
	D+α	4.3	55.5	3.9	94.0	3.9	94.5	8.8	51.6
	CI**_50_**	0.3		0.2		0.2		0.7	
	E_bliss_	53.9%		38.3%		44.1%		36.8%	
Paclitaxel	P	31.2	25.8	23.1	38.9	23.7	25.3	38.2	22.2
	P+α	23.5	24.2	13.03	40.0	12.33	38.5	21.4	25.1
	CI**_50_**	1.2		1.1		1.1		1.03	
	E_bliss_	42.3%		48.7%		51.8%		33.8%	
Vincristine	V	27.1	32.0	28.2	32.9	28.0	38.9	20.5	35.3
	V+α	20.8	32.3	15.7	34.8	13.0	48.0	14.5	42.3
	CI**_50_**	1.2		1.2		1.02		1.02	
	E_bliss_	47.1%		43.6%		60.6%		45.0%	

*: The concentration of α-Mangostin was 10 μmol/L.
