# Supplementary material for: α-Mangostin Suppresses Melanoma Growth, Migration, and Invasion and Potentiates the Anti-tumor Effect of Chemotherapy
Source: Int J Med Sci. 2023 Aug 6;20(9):1220–34. doi: 10.7150/ijms.80940 (PMC10416720; doi:10.7150/ijms.80940)
Supplement: Supplementary file 1 — Supplementary figure and table. [file ijmsv20p1220s1.pdf]

Supplementary Material

1 Supplementary figure

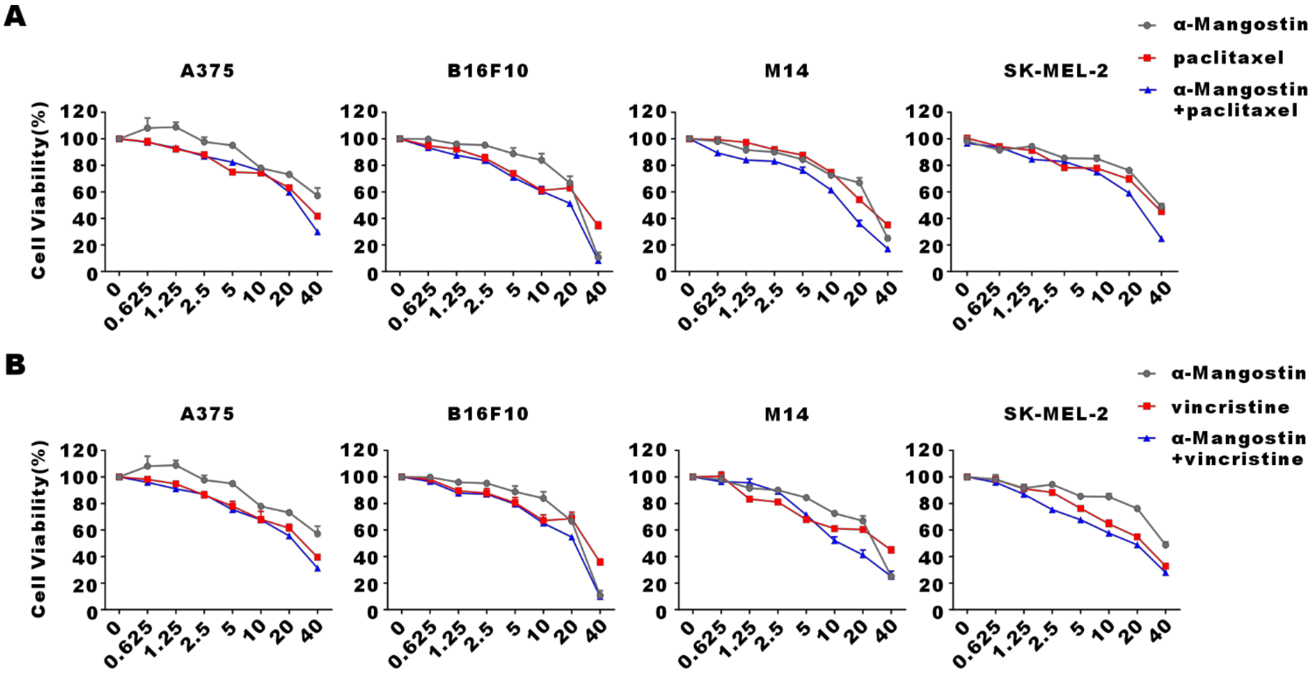

Supplementary Figure 1. Cell viabilities of OS cells after treated with 10 µM α-Mangostin and/or different concentrations of paclitaxel/vincristine for 24 h. Data are presented as the mean ± SEM (n = 5).

2 Supplementary Table

Supplementary Table 1. Primers used in quantitative real-time PCR

Gene	Forward primer (5′ → 3′)	Reverse primer (5′ → 3′)
Human MITF	CTATGCTTACGCTTAACTCCA	TCAAGCCCAAGATTTCCTCA
Mouse MITF	TTATAGTACCTTCTCTTTGCCAGTCC	GTTTATTTGCTAAAGTGGTAGAAAGGTACT
Human RAS	TGGACGGCGAAGTAAAGCATT	AGTGTGACATTGAGGGAGTCG
Mouse RAS	CCTTACGTGAACCTGTGACGAA	AACCTTGAAGCGAACATCGAA

Human PI3K	GGGAGGGCTTTCTTTGTGTA	TCCTGAGCCTGTTTTGTGTCT
Mouse PI3K	TTGAAGAACAATGCCAAACCC	GAATTTCTTTCTCGTTGCCTT
